# ADVANCING OLDER ADULTS’ QUALITY OF LIFE AND INDEPENDENCE WITH FITNESS: INNOVATIVE APPROACHES AND STRATEGIES

**DOI:** 10.1093/geroni/igae098.0295

**Published:** 2024-12-31

**Authors:** Patricia Heyn, James Dowd, James Dowd

**Affiliations:** Chair; Co-Chair; Discussant

## Abstract

This symposium by the Fitness, Exercise, and Wellness (FEW) Interest Group showcases diverse approaches to enhancing well-being and quality of life for older adults through innovative exercise interventions and research methodologies. Dr. Sara Pappa’s work outlines the success of the Northern Virginia Falls Prevention Alliance in deploying community-based evidence-based fall prevention programs, highlighting the effectiveness of academic-community partnerships in implementing and scaling such initiatives. Dr. James Dowd delves into the motivations of older runners, revealing that while health benefits play a role, the habit and joy of running are fundamental factors in their persistence, shedding light on the fortitude of this demographic and the new insights that this research is contributing to the field. Dr. Julie Ries and colleagues address research redundancy in meta-analyses evaluating exercise interventions for older adults with cognitive impairments, advocating for more strategic and ethical research practices to optimize resources and knowledge accumulation. Finally, Dr. Patricia C. Heyn and colleagues present a case study of implementing the 4Ms framework in dementia care in Brazil, emphasizing personalized approaches and the role of technology in maintaining family engagement and improving caregiving quality and connectivity from afar. Together, these innovative presentations highlight the importance of interdisciplinary collaborations, personalized care approaches, and technological innovations in promoting older adults’ functional well-being and autonomy, particularly in the realms of fall prevention, physical activity performance, cognitive health, and dementia care.

